# Continuous deep sedation versus minimal sedation after cardiac arrest and resuscitation (SED‐CARE): A protocol for a randomized clinical trial

**DOI:** 10.1111/aas.70022

**Published:** 2025-04-03

**Authors:** A. Ceric, J. Dankiewicz, J. Hästbacka, P. Young, V. H. Niemelä, F. Bass, M. B. Skrifvars, N. Hammond, M. Saxena, H. Levin, G. Lilja, M. Moseby‐Knappe, M. Tiainen, M. Reinikainen, J. Holgersson, C. B. Kamp, M. P. Wise, P. J. McGuigan, J. White, K. Sweet, T. R. Keeble, G. Glover, P. Hopkins, C. Remmington, J. M. Cole, N. Gorgoraptis, D. G. Pogson, P. Jackson, J. Düring, A. Lybeck, J. Johnsson, J. Unden, A. Lundin, J. Kåhlin, J. Grip, E. M. Lotman, L. Romundstad, P. Seidel, P. Stammet, T. Graf, A. Mengel, C. Leithner, J. Nee, P. Druwé, K. Ameloot, A. Nichol, M. Haenggi, M. P. Hilty, M. Iten, C. Schrag, M. Nafi, M. Joannidis, C. Robba, T. Pellis, J. Belohlavek, D. Rob, Y. M. Arabi, S. Buabbas, C. Yew Woon, A. Aneman, A. Stewart, M. Reade, C. Delcourt, A. Delaney, M. Ramanan, B. Venkatesh, L. Navarra, B. Crichton, A. Williams, D. Knight, J. Tirkkonen, T. Oksanen, T. Kaakinen, S. Bendel, H. Friberg, T. Cronberg, J. C. Jakobsen, N. Nielsen

**Affiliations:** ^1^ Anesthesia and Intensive Care, Department of Clinical Sciences Lund University, Skane University Hospital Malmö Sweden; ^2^ Department of Clinical Sciences Lund, Section of Cardiology Skåne University Hospital Malmö Sweden; ^3^ Faculty of Medicine and Health Technology, Tampere University Hospital Wellbeing Services County of Pirkanmaa and Tampere University Tampere Finland; ^4^ Intensive Care Unit Wellington Hospital Wellington New Zealand; ^5^ Medical Research Institute of New Zealand Wellington New Zealand; ^6^ Australian and New Zealand Intensive Care Research Centre Monash University Melbourne Victoria Australia; ^7^ Department of Critical Care University of Melbourne Melbourne Victoria Australia; ^8^ Department of Anaesthesia and Intensive Care Helsinki University Hospital and University of Helsinki Helsinki Finland; ^9^ The George Institute for Global Health Sydney Australia; ^10^ Royal North Shore Hospital Sydney Australia; ^11^ Critical Care Program The George Institute for Global Health, UNSW Sydney Australia; ^12^ Malcolm Fisher Department of Intensive Care Royal North Shore Hospital St Leonards Australia; ^13^ Critical Care Division and Department of Intensive Care Medicine, The George Institute for Global Health and St George Hospital Clinical School University of New South Wales Sydney Australia; ^14^ Department of Clinical Sciences Lund Lund University Lund Sweden; ^15^ Department of Research, Development, Education and Innovation Skåne University Hospital Lund Sweden; ^16^ Neurology, Department of Clinical Sciences Lund Lund University Lund Sweden; ^17^ Department of Neurology Skåne University Hospital Lund Sweden; ^18^ Department of Neurology and Rehabilitation Skåne University Hospital Lund Sweden; ^19^ Department of Neurology Helsinki University Hospital and University of Helsinki Helsinki Finland; ^20^ Institute of Clinical Medicine University of Eastern Finland Kuopio Finland; ^21^ Department of Anaesthesiology and Intensive Care Kuopio University Hospital Kuopio Finland; ^22^ Department of Clinical Sciences Lund, Anesthesia and Intensive Care Lund University Lund Sweden; ^23^ Department of Anesthesia and Intensive Care Helsingborg Hospital Helsingborg Sweden; ^24^ Copenhagen Trial Unit, Centre for Clinical Intervention Research, The Capital Region Copenhagen University Hospital—Rigshospitalet Copenhagen Denmark; ^25^ Department of Regional Health Research, The Faculty of Health Sciences University of Southern Denmark Odense Denmark; ^26^ Adult Critical Care University Hospital of Wales Cardiff UK; ^27^ Wellcome‐Wolfson Institute for Experimental Medicine Queen's University Belfast Belfast UK; ^28^ Regional Intensive care Unit Royal Victoria Hospital Belfast United Kingdom; ^29^ University Hospitals Bristol and Weston NHS Foundation Trust Bristol UK; ^30^ Essex Cardiothoracic Centre MSE NHSFT Essex UK; ^31^ Anglia Ruskin School of Medicine & MTRC, ARU Chelmsford, Essex United Kingdom; ^32^ Department of Critical Care Guy's and St Thomas NHS Foundation Trust London UK; ^33^ Faculty of Life, Sciences and Medicine, King's College, Intensive Care Medicine, Centre for Human and Applied Physiological Sciences School of Basic and Medical Biosciences London UK; ^34^ Intensive Care Medicine, King's Critical Care King's College Hospital, NHS Foundation Trust London UK; ^35^ Department of Pharmacy and Adult Critical Care, Guy's and St Thomas' NHS Foundation Trust London UK; ^36^ Institute of Pharmaceutical Sciences, School of Cancer & Pharmaceutical Sciences King's College London London UK; ^37^ Critical Care University Hospital of Wales Cardiff UK; ^38^ BartsHealth NHS Trust London UK; ^39^ Department of Critical Care Portsmouth University Hospitals Trust Portsmouth UK; ^40^ Leeds Teaching Hospitals NHS Trust Leeds UK; ^41^ Department of Clinical Sciences, Anesthesia and Intensive Care, Lund University Skåne University Hospital Malmö Sweden; ^42^ Anesthesia and Intensive Care, Department of Clinical Sciences Lund Lund University, Skane University Hospital Lund Sweden; ^43^ Department of Anaesthesiology and Intensive Care Helsingborg Hospital Helsingborg Sweden; ^44^ Department of Operation and Intensive Care Hallands Hospital Halmstad Sweden; ^45^ Department of Intensive and Perioperative Care, Skåne University Hospital Lund University Lund Sweden; ^46^ Department of Anaesthesiology and Intensive Care Medicine, Institute of Clinical Sciences, Sahlgrenska Academy University of Gothenburg Gothenburg Sweden; ^47^ Perioperative Medicine and Intensive Care (PMI) Karolinska University Hospital Stockholm Sweden; ^48^ Department of Physiology and Pharmacology Karolinska Institutet Stockholm Sweden; ^49^ Function Perioperative Medicine and Intensive Care Karolinska University Hospital Stockholm Sweden; ^50^ Department of Clinical Science, Interventionand Technology Karolinska Institute Stockholm Sweden; ^51^ North Estonia Medical Centre Tallinn Estonia; ^52^ Department of Anesthesia and Intensive Care medicine, Division of Emergencies and Critical care Oslo University Hospital Oslo Norway; ^53^ Lovisenberg Diaconal University College Oslo Norway; ^54^ Department of Intensive Care Medicine Stavanger University Hospital Stavanger Norway; ^55^ Department of Anaesthesia and Intensive Care Medicine Centre Hospitalier de Luxembourg Luxembourg Luxembourg; ^56^ Department of Life Sciences and Medicine, Faculty of Science, Technology and Medicine University of Luxembourg Esch‐sur Alzette Luxembourg; ^57^ University Heart Center Lübeck University Hospital Schleswig‐Holstein Schleswig‐Holstein Germany; ^58^ German Center for Cardiovascular Research (DZHK) Hamburg/Lübeck/Kiel Germany; ^59^ Department of Neurology and Stroke University Hospital Tuebingen, Hertie Institute of Clinical Brain Research Tuebingen Germany; ^60^ Charité—Universitätsmedizin Berlin, Department of Neurology Freie Universität and Humboldt‐Universität zu Berlin Berlin Germany; ^61^ Department of Nephrology and Medical Intensive Care Charité—Universitaetsmedizin Berlin Berlin Germany; ^62^ Department of Intensive Care Medicine Ghent University Hospital Ghent Belgium; ^63^ Department of Cardiology, Ziekenhuis Oost‐ Genk Limburg Belgium; ^64^ University College Dublin Clinical Research Centre at St Vincent's University Hospital University College Dublin Dublin Ireland; ^65^ The Australian and New Zealand Intensive Care Research Centre Monash University Melbourne Australia; ^66^ The Alfred Hospital Melbourne Australia; ^67^ Institute of Intensive Care Medicine University Hospital Zurich Zurich Switzerland; ^68^ Institute of Intensive Care Medicine University Hospital Zurich Zurich Switzerland; ^69^ Department of Intensive Care Medicine Inselspital University Hospital Bern Bern Switzerland; ^70^ Klinik für Intensivmedizin, Kantonsspital St. Gallen St. Gallen Switzerland; ^71^ Istituto Cardiocentro Ticino Lugano Switzerland; ^72^ Division of Intensive Care and Emergency Medicine, Department of Internal Medicine Medical University Insbruck Innsbruck Austria; ^73^ IRCCS Policlinico San Martino, Genova, Italy. Dipartimento di Scienze Chirurgiche Diagnostiche Integrate University of Genova Genova Italy; ^74^ Anaesthesia and Intensive Care Pordenone Hospital, Azienda Sanitaria Friuli Occidentale Pordenone Italy; ^75^ First Faculty of Medicine Charles University in Prague, Institute for Heart Diseases Prauge Czech Republic; ^76^ Second Department of Internal Medicine, Cardiovascular Medicine General University Hospital, Wroclaw Medical University Wroclaw Poland; ^77^ Second Department of Medicine, Department of Cardiovascular Medicine, First Faculty of Medicine Charles University in Prague and General University Hospital in Prague Prague Czech Republic; ^78^ King Abdullah International Medical Research Center King Saud bin Abdulaziz University for Health Sciences Riyadh Saudi Arabia; ^79^ Department of Anestesia, Critical Care and Pain Medicine Jaber Alahmad Alsabah Hospital Kuwait City Kuwait; ^80^ Tan Tock Seng Hospital Singapore Singapore; ^81^ Yong Loo Lin School of Medicine National University of Singapore Singapore Singapore; ^82^ Lee Kong Chian School of Medicine Nanyang Technological University Singapore Singapore; ^83^ Intensive Care Unit Liverpool Hospital, South Western Sydney Local Health District Sydney New South Wales Australia; ^84^ South Western Clinical School University of New South Wales Sydney New South Wales Australia; ^85^ The Ingham Institute for Applied Medical Research Sydney New South Wales Australia; ^86^ Liverpool Hospital Liverpool Australia; ^87^ Medical School University of Queensland, Level 9, Health Sciences Building, Royal Brisbane and Women's Hospital Brisbane Australia; ^88^ The George Institute for Global Health, Faculty of Medicine University of New South Wales Sydney Australia; ^89^ Department of Clinical Medicine, Faculty of Medicine, Health and Human Sciences Macquarie University Sydney Australia; ^90^ Critical Care Program, The George Institute for Global Health. Malcolm Fisher Depratment of Intensive Care Medicine, Royal North Shore Hospital. Northern Clinical School, Sydney Medical School University of Sydney Sydney Australia; ^91^ Caboolture and Royal Brisbane and Women's Hospitals Metro North Hospital and Health Service Brisbane Queensland Australia; ^92^ School of Clinical Medicine Queensland University of Technology Brisbane Queensland Australia; ^93^ Critical Care Division, The George Institute for Global Health University of New South Wales Sydney New South Wales Australia; ^94^ Middlemore Hospital Auckland New Zealand; ^95^ Department of Intensive Care Christchurch Hospital Christchurch Central City New Zealand; ^96^ Intensive Care Unit Tampere University Hospital Tampere Finland; ^97^ Department of Anaesthesia and Intensive Care Jorvi Hospital, University Hospital of Helsinki and University of Helsinki Espoo Finland; ^98^ Research Unit of Translational Medicine, Research Group of Anaesthesiology, Medical Research Center Oulu Oulu University Hospital and University of Oulu Oulu Finland; ^99^ OYS Heart, Oulu University Hospital, MRC Oulu and University of Oulu Oulu Finland; ^100^ Anesthesia and Intensive Care, Department of Clinical Sciences Lund Lund University Lund Sweden; ^101^ Intensive and Perioperative Care Skåne University Hospital Malmö Sweden; ^102^ Copenhagen Trial Unit, Centre for Clinical Intervention Research Copenhagen University Hospital—Rigshospitalet Copenhagen Denmark; ^103^ Department of Regional Health Research, Faculty of Health Sciences University of Southern Denmark Odense Denmark

**Keywords:** cardiac arrest, randomized clinical trial, sedation

## Abstract

**Background:**

Sedation is often provided to resuscitated out‐of‐hospital cardiac arrest (OHCA) patients to tolerate post‐cardiac arrest care, including temperature management. However, the evidence of benefit or harm from routinely administered deep sedation after cardiac arrest is limited. The aim of this trial is to investigate the effects of continuous deep sedation compared to minimal sedation on patient‐important outcomes in resuscitated OHCA patients in a large clinical trial.

**Methods:**

The SED‐CARE trial is part of the 2 × 2 × 2 factorial *Sedation, Temperature and Pressure after Cardiac Arrest and Resuscitation* (STEPCARE) trial, a randomized international, multicentre, parallel‐group, investigator‐initiated, superiority trial with three simultaneous intervention arms. In the SED‐CARE trial, adults with sustained return of spontaneous circulation (ROSC) who are comatose following resuscitation from OHCA will be randomized within 4 hours to continuous deep sedation (Richmond agitation and sedation scale (RASS) −4/−5) (*intervention*) or minimal sedation (RASS 0 to −2) (*comparator*), for 36 h after ROSC. The primary outcome will be all‐cause mortality at 6 months after randomization. The two other components of the STEPCARE trial evaluate sedation and temperature control strategies. Apart from the STEPCARE trial interventions, all other aspects of general intensive care will be according to the local practices of the participating site. Neurological prognostication will be performed according to European Resuscitation Council and European Society of Intensive Care Medicine guidelines by a physician blinded to the allocation group. To detect an absolute risk reduction of 5.6% with an alpha of 0.05, 90% power, 3500 participants will be enrolled. The secondary outcomes will be the proportion of participants with poor functional outcomes 6 months after randomization, serious adverse events in the intensive care unit, and patient‐reported overall health status 6 months after randomization.

**Conclusion:**

The SED‐CARE trial will investigate if continuous deep sedation (RASS −4/−5) for 36 h confers a mortality benefit compared to minimal sedation (RASS 0 to −2) after cardiac arrest.

## 1 BACKGROUND AND SIGNIFICANCE

Approximately 700,000 individuals suffer from out‐of‐hospital cardiac arrest (OHCA) annually in Europe and in the United States.^1^, ^2^, ^3^ After resuscitation from cardiac arrest and regaining spontaneous circulation, most cardiac arrest patients remain unconscious and require intensive care treatment.^4^, ^5^ The post‐cardiac arrest period involves many clinical decisions, including ventilator strategy, hemodynamic targets, vasopressor support, organ support, withdrawal of life‐sustaining therapies, and providing patient comfort through sedation and analgesia.^6^, ^7^, ^8^ Temperature management is the only intervention that has been recommended by guidelines to reduce hypoxic–ischemic brain injury and increase the likelihood of a good functional outcome; however, it has limited supportive evidence.^6^

For general intensive care unit (ICU) patients, current guidelines recommend minimizing sedation through daily interruptions of sedative infusions or using a protocol targeting light sedation.^9^, ^10^, ^11^ In post‐cardiac arrest care, short‐acting sedatives and opioids are recommended during targeted temperature management (TTM) to ensure patient comfort and reduce shivering, but with sedation breaks advised only after rewarming.^12^, ^13^ Additionally, approximately one third of cardiac arrest patients have seizures, which could further aggravate the hypoxic brain injury.^14^ Sedation, a powerful anti‐seizure strategy, has been proposed as a potentially brain‐protective and seizure‐prophylactic intervention based on physiological reasoning and some experimental studies.^15^, ^16^, ^17^ Sedation may also support brain protection by enhancing glymphatic system function through inducing non‐rapid eye movement (REM) sleep, which helps clear toxic breakdown products such as β‐amyloid and tau‐protein, potentially reducing the risk of neurodegeneration.^18^, ^19^

While sedation can provide benefits by alleviating discomfort, pain, and anxiety as well as facilitating necessary procedures and treatments, it is important to consider the associated risks. Deeper sedation can compromise circulatory and respiratory functions and increase the risk of adverse effects like pneumonia, delirium, delayed awakening and mobilization, and prolonged ICU stay, which may negatively affect outcome.^20^, ^21^, ^22^, ^23^, ^24^ In addition, sedation interferes with neurological prognostication as it confounds clinical neurologic examination of consciousness and may alter electroencephalography (EEG) patterns.^25^, ^26^, ^27^

Sedation depth is most effectively monitored using clinical sedation assessments such as the Richmond Agitation and Sedation Scale (RASS) a well‐established, validated, and reliable sedation scale for general ICU patients.^28^, ^29^ However, comatose cardiac arrest patients have been excluded in the development of sedation scales to avoid confounding interaction between unresponsiveness due to brain injury and sedation, making the assessment of sedation depth in these patients difficult.^28^, ^29^

In cardiac arrest patients with hypoxic–ischemic encephalopathy, it is unclear whether the potential benefits of sedation outweigh the detrimental effects. In the context of these conflicting arguments for and against deep sedation in the first days following cardiac arrest, there is substantial uncertainty as to optimal clinical practice.

Here, we describe the Sedation after Cardiac Arrest and Resuscitation (SED‐CARE) trial, which is part of the factorial STEPCARE randomized clinical trial. The two other interventions of the STEPCARE trial (temperature and blood pressure) are described separately.

## 2 METHODS

### 2.1 Trial design

The SED‐CARE trial is registered at clinicaltrials.gov (NCT05564754, 2022‐10‐03) as part of the 2 × 2 × 2 factorial STEPCARE trial. The STEPCARE trial protocol was designed following the SPIRIT guidelines, and the trial will be reported according to the CONSORT guidelines.^30^, ^31^, ^32^ The full STEPCARE trial protocol is available at https://stepcare.org/. The STEPCARE trial is a randomized international, multicenter, parallel‐group, investigator‐initiated, superiority trial with three simultaneous intervention arms, considered three separate trials. In the SED‐CARE trial, participants will be randomized to cardiac arrest management with continuous deep sedation for 36 h or minimal sedation. In the two other interventions, participants will be randomized to fever treatment with or without a device and to higher or lower mean arterial blood pressure treatments. Apart from the interventions of the STEPCARE trial, intensive care management will be according to international guidelines and the local practices of each participating hospital.

### 2.2 Inclusion criteria

SED‐CARE will include adults (≥18 years) who experience an OHCA with sustained return of spontaneous circulation (ROSC; 20 min of spontaneous circulation without the need for chest compressions) and who are unconscious, defined as not being able to obey verbal commands (Full Outline of UnResponsiveness [FOUR] score motor response <4) or who are intubated and sedated because of agitation after sustained ROSC.^33^ Participants must not have treatment limitations for intensive care (e.g., a “do not attempt resuscitation” order or a decision not to escalate care) to be included in the trial. Screening will be performed as soon as possible but no later than 4 h after ROSC.

### 2.3 Exclusion criteria

Exclusion criteria will be trauma or hemorrhage (including gastrointestinal bleeding) as the presumed cause of the arrest, pregnancy, suspected or confirmed intracranial hemorrhage, and previous randomization to the STEPCARE trial. Additionally, patients treated with extracorporeal membrane oxygenation (ECMO) prior to randomization will be excluded.

### 2.4 Screening and randomization

Screening will be performed in the emergency room, angiography suite, or the ICU. Clinical investigators at each participating site will be responsible for the screening of all patients who are resuscitated from an OHCA. A screening log will be compiled, including all patients with sustained ROSC admitted to the ICU, to document whether they are eligible for inclusion. Informed consent will be obtained according to national ethical approvals. The reason for the exclusion of screened patients will be documented and reported. Randomization will be performed via a web‐based application to allow for immediate allocation to treatment groups and to ensure allocation concealment and adequate allocation sequence generation. Randomization will be performed with blinded permuted blocks of varying sizes, stratified for trial site.

### 2.5 Intervention

The intervention will begin immediately after randomization by adjusting sedative infusions and administrations to the targeted sedation level. However, if needed, patients can be initially sedated to ensure safe transport, imaging, coronary angiography, and other invasive procedures. The sedation depth will be assessed and determined using the RASS. The RASS score ranges from −5 (un‐arousable) to 0 (alert and calm) to +4 (combative). RASS −4 to −5 is unresponsive to voice and considered deep sedation, while a RASS score of 0 to −3 is a patient responsive to voice and considered light sedation. The RASS score and motor response (defined as obeying commands) will be recorded every 4 h.

In both study groups, multimodal pain management will be adopted, including non‐pharmacological techniques, acetaminophen (paracetamol), and opioids by either continuous or intermittent intravenous infusion. Pain management and treatment for delirium should follow the principles outlined by the Society of Critical Care Medicine (SCCM).^34^ Thus, in both study groups, sedative medications should only be used to achieve the prescribed sedation target, and only after measures to control pain and delirium have been initiated. For patients receiving neuromuscular blocking agents, the level of sedation should be titrated to avoid awareness, as per the treating physician, regardless of the allocation group. After the 36‐h intervention period, the sedation strategy for both groups will be at the discretion of the treating physician as needed for clinical care. Reasons for deviations from the allocated sedation target will be recorded. In accordance with guidelines, short‐acting sedatives such as propofol should be preferred to benzodiazepines (by either continuous infusion or bolus dosing), but the type and dose of sedatives used to achieve the target RASS will be at the discretion of the treating physician. Occasionally, patients may have a particular clinical indication for a benzodiazepine‐based sedation regimen: for example, complementing very high rates of propofol infusion or marked hemodynamic instability. In such cases, the requirement for continuing benzodiazepine use (vs. an alternative) should be reassessed continuously.

#### 2.5.1 Continuous deep sedation for 36 h

For patients randomized to continuous sedation, a continuous infusion of a short‐acting sedative agent (such as propofol) should be started at randomization to target deep sedation (RASS −4 to −5) and continued until 36 h after randomization. During the first 36 h, this infusion should be increased if the patient becomes rousable, with a RASS target of −4 to −5 until 36 h after randomization. After 36 h, the sedation goal should be according to the treating clinician and continued until the time of liberation from mechanical ventilation, at which time all sedative medications should be discontinued as soon as judged safe.

#### 2.5.2 Minimal sedation

In patients randomized to minimal sedation, sedative agents shall not be used unless needed for clinical care. During the first few hours of post‐cardiac arrest care, patients may require sedation to facilitate safe transfers, imaging, and invasive procedures, but weaning from sedatives should be performed as early as possible, ideally within 6 h of randomization if not at the time of ICU admission. Patients will be continuously assessed for possibilities for extubation immediately after admission to the ICU according to local criteria. If the patient is alert, obeys commands, and is otherwise stable, the patient may be extubated. A patient who does not fulfill criteria for safe extubation should remain intubated and receive minimal sedation as needed for clinical care.

Pain assessment and adequate analgesia are required before providing sedation, which is provided only if analgesia alone is insufficient in ensuring safety in clinical care. If sedation is needed, it should be targeted at the lightest possible level that enables safe treatment and adequate patient comfort. The sedation target should be RASS 0 to −2, unless there is a clinical indication for deeper sedation, in which case a deeper sedation target is acceptable. Although anti‐seizure medications should be the primary treatment of refractory status epilepticus and myoclonus, deeper sedation may be used in the minimal sedation arm when necessary to manage clinical situations such as refractory status epilepticus, myoclonus, severe hypoxemia, or confirmed or suspected raised intracranial pressure.

### 2.6 Changing sedation

The clinical situation may require continuous sedation to be started in a patient in the minimal sedation group. This is at the discretion of the treating physician. If continuous sedation is started within the first 36 h, the reason for this will be recorded and classified as follows:

Was continuous sedation started within 36 h of randomization?

- No
- Yes—to facilitate general intensive care
- Yes—for seizures

### 2.7 Shivering

Shivering will be recorded according to the bedside shivering assessment scale (BSAS) on Day 1–3.^35^ The treatment goal for shivering will be to maintain a BSAS score of 0 or 1. To ensure adequate control of shivering, local protocols should be followed. Interventions to minimize shivering will be decided by the treating clinician but might include paracetamol, magnesium, buspirone, increased sedation, and administration of a non‐depolarizing neuromuscular blocking agent.

### 2.8 General Intensive care

General intensive care, including management of respiration, metabolic disturbances, ulcer prophylaxis, deep venous thrombosis prophylaxis, and other aspects of intensive care, should be delivered similarly in all allocation groups and according to local protocols at the discretion of the treating physicians. Cardiac interventions will also be guided by local protocols. However, participating centers will need to have access to around‐the‐clock invasive management, either on‐site or at a nearby hospital, which is also part of the trial. Cardiac catheterization (coronary angiography) should not be delayed by the trial interventions. Apart from the interventions, adhering to international and national guidelines for post‐resuscitation care is recommended.

### 2.9 Blinding

The clinical team responsible for the immediate care of the participant will not be blinded to the study interventions due to the inherent difficulty in blinding the interventions (sedation, temperature, and arterial blood pressure). Measures will be taken to ensure that allocation information will only be disseminated within the immediate group of health care workers responsible for patient care. A blinded physician will make a first prognostic evaluation of the participant 72 h after randomization and make a statement on neurological prognosis (for details, see below).

Participants, their legal representatives, and family will only be informed that the patient has been part of the trial but not the allocation group. The outcome assessors, prognosticators, statisticians, the data safety monitoring committee (DSMC), members of the steering committee, and authors of the manuscript will be blinded to treatment allocation. The intervention groups will be coded as “X” and “Y” Two abstracts will be prepared, one assuming X is the experimental group and Y is the control group—and one assuming the opposite. The author group must approve conclusions before the randomization code is broken.

### 2.10 Prognostication and withdrawal of life‐sustaining therapies

The SED‐CARE trial will employ a conservative and strict protocol for neurological prognostication according to the European Resuscitation Council (ERC) and European Society of Intensive Care Medicine (ESICM) recommendations (see supplement).^6^, ^36^ Prognostication will be performed on all participants who are not awake and obeying verbal commands and who are still in the ICU at 72 h after randomization. Prognostication must be sufficiently delayed, ensuring that any lingering effects of sedative agents will not affect the assessment. Prognostication will be made by a physician experienced in neuro‐prognostication after cardiac arrest, blinded to treatment allocations. The blinded external physician will not make specific recommendations about the withdrawal of life‐sustaining measures.

Presumed poor functional outcome will not justify the withdrawal of life‐sustaining therapies (WLST) prior to prognostication. Life‐sustaining therapies may only be withdrawn before protocolized prognostication in the following situations: if information on a pre‐existing advanced care directive or an advanced medical comorbidity (e.g., generalized malignant disease) that prohibits continuation of care becomes available after inclusion in the trial, or if continuation of care is considered unethical due to irreversible multi‐organ failure. Brain death, established according to local legislation, will be defined as death and not WLST. See supplemental material for a detailed description of neurological prognostication and WLST.

### 2.11 Follow‐up

Long‐term outcomes will be assessed and recorded during a telephone follow‐up at 30 days and during a physical visit or a telephone/virtual meeting 6 months after randomization. The blinded outcome assessor may be an occupational therapist, physician, research nurse, psychologist, or another health care professional. The central follow‐up coordinating team will provide outcome assessors with detailed guidelines and study‐specific training. More detailed outcomes will be collected in an extended follow‐up sub‐study at selected sites, including, for example, cognitive function, societal participation, and family impact. This sub‐study is described elsewhere.

### 2.12 Outcome measures

The primary and secondary outcomes will be assessed 6 months after randomization. The primary outcome will be all‐cause mortality. Secondary outcomes will be the proportion of participants with a poor functional outcome defined primarily as a score of 4–6 (moderately severe disability, severe disability or dead) reported by the structured modified Rankin Scale (mRS, range 0–6 with higher scores indicating a worse outcome).^37^ If an mRS score cannot be assigned, patients will be categorized based on whether they are dependent on others for basic activities of daily life (need of assistance with, for example, moving indoors, eating, dressing, taking care of personal hygiene), similar to an mRS score of 4–6 but without the detailed information that is needed to separate outcomes between categories. Other secondary outcomes will include the proportion of patients who died or had a predefined serious adverse event in the ICU and patient‐reported overall health by using the EQ visual analogue scale (EQ VAS), a part of the EQ‐5D.^38^

Exploratory outcomes will be ventilator‐free days within the first 30 days, hospital‐free days within the first 30 days, mRS (ordinal scale), time‐to‐event and win ratio (dead versus alive), all steps on the mRS scale, safety event, and detailed information from the EQ‐5D‐5L.

### 2.13 Adverse events

It is recognized that the intensive care patient population will experience several common aberrations in laboratory values, signs, and symptoms due to the severity of the underlying disease and the impact of standard therapies. Intensive care patients will frequently develop life‐threatening organ failure(s) unrelated to study interventions, despite optimal management. Therefore, consistent with established practice in academic ICU trials,^39^ events that are part of the natural history of the primary disease process or expected complications of critical illness will not be reported as adverse events in this study. All adverse events that are potentially causally related to the study intervention or are otherwise of concern in the investigator's judgment will be reported and reviewed by the management committee and DSMC. A number of specified serious adverse events (SAEs) (as described below) are captured in the trial case report form and will not be separately reported as SAEs.

Only predefined SAEs (Table 1) and any unexpected SAEs will be reported by the investigator to avoid overreporting and to maximize the probability of finding true and important differences. We predefined SAEsbased on known and plausible harms of sedation management.

**TABLE 1:** Definition of specific serious adverse events.

Serious adverse event	Definition
Sepsis or septic shock	Sepsis‐3 criteria^40^
Arrhythmia requiring defibrillation, cardioversion or chest compressions	Arrhythmia requiring defibrillation, cardioversion or chest compressions
Venous thromboembolism	Venous thromboembolism confirmed by imaging. Thrombi related to intravascular cooling devices should be classified as deep vein thrombosis
Moderate or severe bleeding	GUSTO criteria (global utilization of streptokinase and tissue plasminogen activator for occluded coronary arteries)^41^
Reintubation	
Non‐planned extubation	

### 2.14 Rationale for chosen outcomes

All‐cause mortality was chosen as the primary outcome to ensure an unbiased assessment and avoid competing risks. We will use the mRS scale to evaluate functional outcome.^42^ The mRS scale is increasingly used in cardiac arrest research and is currently recommended by the Core Outcome Set for Cardiac Arrest (COSCA) and the International Liaison Committee on Resuscitation (ILCOR) consensus statement for measuring functional outcome after cardiac arrest.^37^ The primary analysis will be a binary analysis, with the mRS dichotomized to 0–3 (none to moderate disability) versus 4–6 (severe disability or death), as this dichotomization separates patients that are non‐dependent from patients that are dependent on others in basic activities of daily living. This dichotomization is also previously used in cardiac arrest trials.^37^, ^42^

The EuroQol‐visual analogue scale (EQ‐VAS) included as a part of EQ‐5D‐5L will be used to measure a patient‐reported outcome of overall health status. This instrument was chosen since it is easy to use, has shown evidence to be a valid measure in many situations, and could be used as a proxy report if necessary.^38^ We will measure possible harmful effects of the intervention by predefined SAEs that are most common and plausibly related to the intervention. A more detailed description of the rationale for chosen outcomes and the exploratory outcomes is available in the supplement.

### 2.15 Factorial design and intervention interactions

Factorial trials have the inherent risk of potential interactions between trial interventions in both physiological and patient‐centered outcomes.^43^ This trial is conducted under the assumption that there is no interaction between the interventions on the present trial's outcomes. The SED‐CARE trial intervention has potential physiological interaction with the temperature and mean arterial pressure intervention of the STEPCARE trial. Targeting deep sedation may cause additional vasodilation and a potential effect on cardiovascular function; consequently, lower blood pressure may therefore affect the achievement of the allocated mean arterial pressure (MAP) target and body temperature. However, previous pilot studies suggest a higher MAP target is achievable in most patients.^44^, ^45^ Additionally, sedation may interact with the temperature intervention, as sedative agents such as propofol may promote heat loss through vasodilation and might also directly impair hypothalamic temperature regulation.^46^ However, no evidence suggests that this interaction could affect the outcomes being assessed. If higher doses of sedatives/deeper levels of sedation, inotropic/vasopressor support, or external cooling are required because of between‐group interactions, differential adverse effects of these interactions are theoretically possible. On the other hand, in the minimal sedation group, some patients who wake up and are extubated early may be discharged from the ICU early, which may affect the duration of the other interventions. The DSMC will monitor the trial during its conduct to identify possible interaction effects on outcomes, focusing on patient safety. For more specific situations of intervention interaction, see Supporting Information S1

### 2.16 Co‐enrolment in other trials

Study participants may be included in any observational trial that does not affect protocol adherence in the STEPCARE trial. We will assess co‐enrolment suggestions based on the Spice‐8 co‐enrolment guidelines.^47^ Unless there are clear conflicts between trial interventions, co‐enrolment in other trials will be possible. The STEPCARE management committee will assess co‐enrolment on a case‐by‐case basis.

### 2.17 Data collection and management

Individual patient data regarding background characteristics, clinical features, and laboratory results will be obtained from medical records, ambulance services, and relatives. Detailed data, including neurological status, body temperature, blood pressure values, and doses of vasoactive and sedative medications, will be collected. Detailed information on collected data is described in the supplement. Data will be entered into a web‐based electronic case report form (eCRF) by site personnel. The software for the eCRFs is provided by Spiral, New Zealand, but the storage server for the trial database will be handled by the trial's coordinating team.

### 2.18 Sedation specific data collection

The total cumulative dose of the following medications during the intervention up to 72 h post‐randomization will be recorded: noradrenaline, propofol, midazolam, remifentanil, sufentanil, fentanyl, dexmedetomidine, paracetamol/acetaminophen, oxycodone, and morphine. Additionally, data on propofol dose (mg/kg/h), dexmedetomidine dose, noradrenaline dose (mcg/kg/min), and midazolam infusion (yes/no) will be collected every 4 hours during the ICU stay up to 120 h.

### 2.19 Sample size and power estimation

The sample size estimation is based on a 60% mortality in the control arm and 54.4% mortality in the intervention arm at 6 months, referring to the results of the TTM1 trial,^48^ the TTM2 trial,^49^ and the International Cardiac Arrest Registry (INTCAR).^50^ To demonstrate a relative risk of 0.91 with 90% power at a significance level of 0.05, 1639 participants are required in each group for a total of 3278 participants. In the TTM2 trial, loss to follow‐up was approximately 2%, and we expect a similar loss to follow‐up in the STEPCARE trial.^49^ Therefore, the sample size was increased by 6.8% to 3500 participants; 1.8% of the increment is considered to account for loss to follow‐up and, as a pragmatic choice, 5% is considered to account for possible interactions between interventions on patient‐centered outcomes. The sample size calculation corresponds to a relative risk reduction of 9.3% and an absolute risk reduction of 5.6%, which is a clinically relevant and realistic treatment effect. For the secondary outcomes, there is an estimated power of 91% to detect a relative risk reduction of 0.9 for poor outcome (mRS 4–6), a power of >90% to detect a difference of 5 points on the EQ‐5D‐5L VAS scale, and a power of 91% to detect a relative risk reduction or increase of 10% for the predefined SAEs (Table 1).^51^

### 2.20 Statistical analyses

All analyses will be conducted according to the intention‐to‐treat principle and adjusted for ‘site’ and the allocated intervention in the two other trials of the factorial STEPCARE trial. Dichotomous outcomes will be presented as proportions of participants with the event, and relative risks with 95% confidence intervals. Continuous data will be presented as means and standard deviations for each group, with 95% confidence intervals for the means of the groups and the differences between the means of the groups. Count data will be presented as means, mean differences, and 95% confidence intervals or medians, interquartile ranges, and 95% confidence intervals depending on the observed distribution. Dichotomous outcomes will be analyzed using a mixed effects generalized linear model, continuous outcomes using a mixed effects linear regression model, and count data using the Wilcoxon test. Mock tables, curves, and graphs presenting characteristics of the participants, sedation depth and motor response results, and separation of sedation level between groups and sedative drugs and dose are provided in the supplement. A detailed statistical analysis plan will be published separately.

### 2.21 Subgroup analysis

The following subgroup analyses will be performed:

- Age (< median or ≥median)
- Sex (male/female)
- Bystander cardiopulmonary resuscitation (yes/no)
- Initial rhythm (shockable versus non‐shockable)
- Time to ROSC (< median or ≥median)
- Circulatory status on admission (presence or absence of circulatory shock diagnosed by the treating physician)
- Baseline risk of poor functional outcome (Miracle2‐score: low risk [0–2], medium *risk* [3‐5], and high *risk* [6‐10]).^52^
- Presumed cause of cardiac arrest at randomization (cardiac vs. others), and
- Diagnosed with chronic hypertension (yes/no).

### 2.22 Sub‐studies

The main sub‐studies of the STEPCARE trial include a biomarker study, an acute kidney injury study, a neuroprognostic study, intensive care monitoring studies, and an extended follow‐up study. Separate protocols will be published for these sub‐studies. Additional sub‐studies will be presented on the STEPCARE trial webpage (www.stepcare.org) and the protocols for these will be published separately.

### 2.23 Ethics and informed consent

Ethics application will seek approval for a delayed written consent process, since the intervention must be regarded as an emergency procedure and must be started as soon as the participants are admitted to hospital. Participants regaining consciousness will be asked for written consent as soon as they are able to make an informed decision. It is of importance that ethical approval contains a request to use data also for deceased participants, to avoid survival bias. The consent process will vary from site to site and will align with local ethical approvals, national laws, and the Declaration of Helsinki.^53^

### 2.24 Data and safety monitoring and interim analysis

The Charter for the DSMC of the STEPCARE trial describes the role and function of the DSMC. The primary focus of the DSMC is the monitoring of the safety and efficacy of the interventions and the overall conduct of the trial to guard the interests of the trial participants. The first interim analysis was conducted after the enrollment of 500 participants, with the recommendation from the DSMC to *continue the trial as planned*. The schedule of further interim analyses will be decided by the DSMC, but a minimum of three interim analyses will be conducted. The DSMC will arrange for an independent statistician to conduct a blinded interim analysis. The DSMC can request the unblinding of data if required. The survival and safety parameters are provided for the DSMC for the conduct of the interim analyses. Lan‐DeMets group sequential monitoring boundaries will be used as the statistical limit to guide recommendations regarding the early termination of the trial.^54^ Interventions of the STEPCARE trial will not be stopped for futility. The DSMC may recommend stopping or pausing the SED‐CARE trial, or the entire STEPCARE trial if:

- Group difference in the primary outcome measure is found in the interim analysis according to predefined stopping rules;
- Group difference in SAEs is found in the interim analysis.
- Evidence of an interaction influencing outcomes; or
- Results from other studies show benefits or harms associated with one of the allocation arms.

It is the steering group's decision whether the trial should be stopped.

### 2.25 Patient group involvement

We followed the COSCA, developed in collaboration with ILCOR, which involved patient representatives to facilitate the selection of patient‐centered outcomes.^37^, ^55^ Patient organizations in Sweden and Australia were involved in the design phase of the STEPCARE trial.

### 2.26 Trial status and timeline

Randomization began in August 2023 and trial sites have been added gradually. The last 6 months follow‐up will be performed presumably during 2026–2027. The results from each intervention and sub‐study will be reported separately. The initial publications for each STEPCARE trial will include results of primary and secondary outcomes.

## 3 DISCUSSION

The aim of this trial is to investigate the effects of deep sedation compared to minimal sedation on outcomes in resuscitated OHCA patients. By investigating these two approaches, we aim to understand the potential benefits and risks associated with each strategy, including their impact on mortality, functional outcome, the occurrence of serious adverse events, and patient‐reported overall health. The findings from this trial have the potential to provide valuable insights in the development of sedation protocols tailored to the needs of cardiac arrest patients, ultimately improving their overall care, outcomes, and optimizing resource utilization. The SED‐CARE trial is part of the larger STEPCARE trial, which also investigates the effects of mean arterial pressure and fever treatment with or without a temperature control device on recovery after cardiac arrest in a factorial fashion.

### 3.1 Rationale for sedation after resuscitation from cardiac arrest

The implementation of hypothermia and temperature management over 20 years ago has led to the routine provision of sedation in post‐cardiac arrest care, with deep sedation commonly used in cardiac arrest trials ever since. However, there are limited data on sedation strategies available to guide clinicians in determining the optimal level of sedation for these patients. Considering the risks of sedation, which may affect circulation and ventilation, as well as its potential benefits and neuroprotective effects is crucial. Furthermore, sedation may interfere with neurological prognostication, confounding the evaluation of consciousness, which could significantly impact patient outcomes through premature withdrawal of treatment based on false pessimistic assessments. Thus, investigating the optimal sedation strategy after cardiac arrest and its effects on post‐cardiac arrest care is essential. The results from the SED‐CARE trial will contribute to future recommendations on sedation practices and provide evidence for optimizing sedation during post‐cardiac arrest care.

### 3.2 Potential consequences of this trial

The minimal sedation group in this trial may experience adverse events including anxiety, discomfort, pain, non‐planned extubation, and development of post‐traumatic stress disorder, potentially contributing to impaired neurological function. On the other hand, the deep sedation group may face consequences such as prolonged mechanical ventilation, immobilization leading to venous thromboembolism, infections causing sepsis, arrhythmias, and possible need for reintubation and prolonged intensive care stay.^20^, ^21^, ^22^, ^23^ The lingering sedation in the deep sedation group may interfere with neurological prognostication.^25^, ^26^ In clinical practice, patients who have experienced cardiac arrest are often managed with deep sedation, similar to the deep sedation group in this trial, while those in the minimal sedation group will be managed like any non‐cardiac arrest ICU patient. Data will be collected to gain a better understanding of these outcomes throughout the trial.

### 3.3 Strengths and limitations

Strengths of the SED‐CARE trial include its large sample size, the broad inclusion criteria, and the predefined detailed methodology leading to results with a low risk of bias. The sample size enables detection of small relative risk reductions and comparisons between a variety of cardiac arrest patient subpopulations that may benefit from different sedation targets. Our choice of patient‐centered outcomes, together with the blinding of outcome assessors, prognosticators, the steering group, author group, statisticians, and the trial coordinating team, represents significant strengths.

Interactions between the blood pressure, sedation, and temperature strategies, with an effect on patient‐centered outcomes, are a possibility and must be considered a limitation. This risk is implicit in all factorial trials. We have designed the study assuming that there is no interaction on the primary, secondary, and exploratory endpoints with the three strategies. Importantly, it is essential to acknowledge that the focus of this trial lies in investigating the targeted sedation depth and not in the assessed sedation depth. This clarification underscores the independence of the trial's treatment allocations and potential interaction effects. Thus, we will not be able to avoid the risk of interactions between interventions because it is an inherent feature of factorial trials. However, we initially deemed the risk of interactions to be minimal. Following calculation of the sample size, we have allowed for a small increment of the sample size (6.8%) to allow for loss to follow‐up and a small interaction effect.

This trial may have a potential weakness in this context, as we include severe critical illness and severely brain‐injured adult critically ill patients admitted to the ICU. As a result, both groups of patients may have deep sedation scores or scores close to deep sedation on the RASS regardless of sedatives administered, making the distinction between the groups less clear due to the severity of illness and brain injury. Additionally, patients with seizures or temperature control devices started in minimal sedation may need increased sedation, also making the separation between groups less clear. This limitation could impact the trial's ability to detect a difference in outcomes, leaving the question unanswered. However, it is important to distinguish that the aim of this trial is to investigate the intervention of targeted sedation levels. To mitigate these challenges, we plan to conduct sensitivity analyses to explore subgroups of patients that may be affected by these possible confounders.

Having open‐label sedation targets is another limitation in this trial. However, measures have been taken to minimize their possible impact on the results and strategies to ensure that we are kept blinded, by blinding the steering group, authors of the manuscript, outcome assessors, prognosticators, statisticians, and the DSMC.

## 4 CONCLUSION

The SED‐CARE trial aims to investigate if continuous deep sedation for 36 h confers a benefit compared to minimal sedation after cardiac arrest on six‐month mortality in adults who are comatose following resuscitation from out‐ofof‐hospital cardiac arrest. This trial aims for a large sample size and will investigate sedation targets in a broad population of cardiac arrest patients. We anticipate that results from this trial will guide future recommendations on sedation management after cardiac arrest. The SED‐CARE trial is part of the large international factorial STEPCARE trial that will also investigate the blood pressure targets and fever treatment after cardiac arrest.

## AUTHOR CONTRIBUTIONS

A. Ceric drafted the manuscript. All other authors contributed to the study design and critically revised the manuscript. All authors approved the final version.

## FUNDING INFORMATION

The trial is funded by The Swedish Research Council, ALF‐project funding within the Swedish Health Care, The Academy of Finland (341277), The Sigrid Jusélius Foundation (Finland) (8137), Hospital district of Helsinki and Uusimaa (Finland), Medicinska Understödsföreningen Liv och Hälsa (Sweden), Medical Research Future Fund (Australia) (199449), Health Research Council of New Zealand, and the Clinical Research Programme Directorate of Health Ministry of Health and Social Security (Luxembourg).

## Supporting information


**Data S1.** Supporting Information.

## Data Availability

Research data are not shared.
